# Plasma endothelial protein C receptor influences innate immune response in ovarian cancer by decreasing the population of natural killer and TH17 helper cells

**DOI:** 10.3892/ijo.2013.2021

**Published:** 2013-07-18

**Authors:** DALEL AZZAZENE, HAMDA AL THAWADI, HALEMA AL FARSI, SAMAHER BESBES, CAROLINE GEYL, SHAHSOLTAN MIRSHAHI, JULIA PARDO, ANNE MARIE FAUSSAT, SORIA JEANNETTE, AMU THERWATH, ERIC PUJADE-LAURAINE, MASSOUD MIRSHAHI

**Affiliations:** 1 National Institut for Medical Research (INSERM), Cordeliers Research Center (UMRS 872), University of Pierre and Marie Curie and University of Paris Descartes;; 2 Hôtel Dieu Hospital, Paris, France

**Keywords:** endothelial protein C receptor, innate immune response, ovarian cancer, natural killer cells, T helper cells

## Abstract

In spite of the growing importance of endothelial protein C receptor/active protein C (EPCR/aPC) in tumor biology, their impact on immunological homeostasis remains largely unexplored. The objective of this study was to assess whether soluble plasma endothelial protein C receptor (sEPCR), which is a regulator of circulating aPC, is involved in innate immune response in cancer patients. In the Ovcar-3 ovarian cancer line, the role of aPC in secretion of cytokines was analyzed. In parallel, in 33 patients, with a diagnosis of ovarian epithelial cancer, sEPCR was quantified, blood immune cell phenotypes were determined by flow cytometry and plasma cytokines were evaluated using a protein array. Spearman’s rank correlation coefficients (r) and coefficient significance was determined by a statistical hypothesis test (α=0.05). Our results show that i) aPC induced the secretion of several cytokines in Ovcar-3 cells; ii) 61% of patients exhibited a concentration of plasma sEPCR well above the baseline (normal plasma level, 100±28 ng/ml); iii) comparing immune cell phenotypes in patients having a normal level of sEPCR with those having a high level of sEPCR, it was found that sEPCR levels were correlated with high intensity of cells expressing CD45ra, CD3, CD8, CD25 and low intensity of cells expressing CD56 (NK cells), CD294 (TH2 cells), IL-2, IL-10, IL-17a (TH17 cells), IL-21 (TH21 cells) and CD29 markers (r ≥0.60); and iv) high levels of sEPCR correlate with high levels of plasma bioactive proteins such as insulin-like growth factor-2 (IGFII), IL-13rα, macrophage inflammatory protein (MIP1α) and matrix metalloproteinase-7 (MMP-7) that have already been proposed as biomarkers for ovarian cancer and particularly those with poor prognosis. In conclusion, sEPCR produced by ovarian cancer cells, by modulating circulating aPC, influences the secretory behavior of tumor cells (cytokines and interleukins). Consequently, sEPCR in turn acts on the innate immune response by decreasing effector cells such as natural killer and T helper cells (TH2, TH17 and TH21).

## Introduction

Endothelial protein C is an important regulator of homeostasis in addition to its involvement in the systemic response to acute inflammation. It is known that circulating protein C zymogens, secreted by liver, binds to endothelial protein C receptor (EPCR) with high affinity and stimulates its activation via the thrombin-thrombomodulin complex. The activated protein C (aPC), together with its cofactor protein S, degrades factors Va and VIIIa and thereby interferes with thrombin generation and inhibits the coagulation cascade ( 1 – 3 ) . EPCR exists as membrane bound as well as free soluble form (sEPCR). sEPCR, infact, can regulate the quantity of circulating aPC ( 4 , 5 ) .

EPCR is a type 1 transmembrane glycoprotein that shares considerable homology with the major histocompatibility complex ( 6 ) . EPCR is known to be constitutively released in the plasma in a free soluble form as a result of proteolytic cleavage. Soluble EPCR has the ability to trap free aPC, thereby depriving the latter of its anticoagulant function (within the surrounding environment) ( 4 – 6 ) . The shedding of EPCR is known to be regulated by IL-1β (interleukin), TNF-α, endotoxin, and via the MAP kinase signaling pathways in human vascular endothelial cell line (HUVEC) ( 7 ) and by the presence of EPCR A3-haplotype homozygosis ( 8 ) .

It is known that in severe sepsis aPC is also involved in preventing thrombosis due to its anticoagulant action ( 9 ) . A drop in levels of protein C in severe sepsis is in fact always associated with poor prognosis ( 10 ) . Several studies have demonstrated the anticoagulant and pro-fibrinolytic nature of aPC and also its involvement in inflammation ( 11 ) . The involvement of certain signaling pathways such as NF-κB ( 12 ) and WNT ( 13 ) were also revealed. On the other hand, the influence of aPC on the immune system is far from clear today.

With the recent appearance of an increasing number of publications, the interest in EPCR/aPC, in relation to tumor biology, is gaining momentum ( 14 – 17 ) . In a previous study, we demonstrated that several solid tumors express EPCR and that sEPCR in patients with ovarian cancer could be a biomarker of cancer expansion ( 18 ) . In addition sEPCR was proposed by us to be a biomarker of cancer associated hypercoagulability in human hematologic malignancies ( 19 ) .

The increase in plasmatic level of sEPCR in cancer patients leads to increased sequestration/capture and use of protein C. This could affect or modulate thrombotic events and immune inflammatory response. As of present, the question of how sEPCR is involved in tumor immunology, however, remains unclear. On the basis of our present findings we are inclined to think that besides being a biomarker of cancer expansion and cancer associated hypercoagulability ( 18 , 19 ) sEPCR (a regulator of circulating aPC) could be involved in the innate immune response in ovarian cancer patients.

## Materials and methods

### 

#### Reagents

Reagents were obtained from following sources: primary antibody AF2245 against the EPCR (R&D Systems, Minneapolis, MN, USA); primary antibody ATAP2 against PAR-1 (Invitrogen, Carlsbad, CA, USA); biotinylated anti-rabbit, anti-mouse and anti-goat IgG, streptavidin-fluorescein conjugate (Amersham, Buckinghamshire, UK); rabbit anti-goat HRP (DakoCytomation, Glostrup, Denmark); human recombinant aPC (Lilly, Suresnes, France).

For phenotyping of immuno-inflammatory cells we used FITC-anti-CD29, PE-anti-CD30, ECD-anti-CD8, PC5-anti-CD25, PC7-anti-CD20, APC-anti-CD14, APC AF700-anti-CD45, APC AF750-anti-CD16, FITC-anti-CD31, PE-anti-CD294, ECD-anti-CD45RA, PC5-anti-CD146, PC7-anti-CD4, APC-anti-CD56, APC-AF700-anti-CD10, APC-AF750-anti-CD3 (all from Beckman Coulter, Roissy CDG, France). FITC-anti-CD94, PE-anti-HLA-G, APC-anti-ROR-G, PercPcy 5.5-anti-CD161. FITC-anti-IL2, PE-anti-IL21, PC5-anti-FOXP3, PercpCy 5.5-anti-IL17A, APC-anti-TGFβ (transforming growth factor), PE-anti-CD133, PE-anti-IL10 (all from eBiosciences, Montrouge, France). The 174 and 100 biotin label-based human antibody array from RayBioTech (Clinisciences, Montrouge, France) were used.

#### Cells

Ovarian cancer cell lines (Ovcar-3, Skov-3, ATCC) were used. Cells were cultured in DMEM medium containing 10% fetal calf serum, penicillin (50 U/ml), and streptomycin (50 *μ* g/ml) and incubated in a humidified atmosphere containing 5% CO _2_ at 37°C, as recommended by the supplier (PAA Laboratories Inc, Etobicoke, ON, USA).

### Plasma and blood mononuclear cells

#### Plasma samples

Patients were aged >18 years, with a histological proven diagnosis of epithelial cancer of the ovary with asymptomatic disease in progression (detected by increase of CA125 levels according to Gynecologic Cancer Intergroup criteria). Ovarian tumors of low malignant potential or non-epithelial ovarian or mixed epithelial/non-epithelial tumors were excluded. Blood samples from patients (n=33) were obtained after informed consent, in accordance with the rules of the revised Helsinki protocol and with Articles L. Al 1121-1 1, L. 1221-8 and L. 1221-8-1, and following the Code of Public Health (CSP), the plasmas were provided to us with a label depicting a code number without the name of the patient. All participants provide their written consent to participate in this study and the ethics committees CPP of Ile de France-1 (regional committee) and National Agency for the Safety of Drugs and Health Products (ANSM national committee) approved this consent procedure and this study.

#### Mononuclear cells

PBMCs were collected from 33 cancer patients at the Hospital Hôtel-Dieu and mononuclear cells were separated by Ficoll gradient centrifugation and then stored in 200 *μ* l of DMSO (10%)/FCS 90% at −80°C.

Phenotyping of immuno-inflammatory cells by immunocytochemical analysis. Flow cytometry was carried out using the 30 antibodies as described in Reagents section.

Mononuclear cells were labeled with several antibodies bound to different fluorescent agents. The controls were performed using corresponding isotype antibodies. The cytometer used was a type analyzer LSRII (BD Bioscience, Le Pont de Claix, France) to 9 colors and 4 lasers.

The antigens, detected on appropriate cells, were identified and analyzed by ‘DAVID Gene Concept’ which is a functional annotation tool. It allows ranking functional categories of sets of genes and unraveling new biological processes associated with cellular functions. The results are expressed as percentage of cells for each blood sample. The analysis was done on the area bound P1 representing lymphocytes. The protocol used in this project was developed using samples obtained from five healthy females (aged 25–50 years).

#### Soluble EPCR-ELISA assay

Soluble EPCR in plasma fluids was measured using Asserachrom sEPCR immunoassay as recommended by the commercial supplier (Diagnostic Stago, Asnieres sur Sein, France).

### Cytokine array

#### The effect of APC on the ovarian cancer cell line

To analyze the pleiotropic role of APC, we examined the supernatant of Ovcar cells using a protein cytokine array (RayBio ^®^ Human Cytokine Antibody). This technique is based on the principle of ‘sandwich immunoassay’. It comprises essentially of screening, in duplicate, 174 different membrane-coupled anti-cytokines along with appropriate controls (experiments repeated 3 times).

Ovcar-3 cells (10 ^6^ cells per ml) were incubated in presence (or not) of APC (200 ng/ml ^−1^ ) in DMEM without fetal calf serum at 37°C in a humidified atmosphere of 5% CO _2_ for 24 h. Supernatants containing cytokines were retrieved and the cytokines were allowed to couple with their specific antibodies previously immobilized on membranes. Membranes were saturated for 2 h at room temperature with bovine serum albumin (BSA). Incubation of array membranes with supernatants (along with controls) was carried out overnight at 4°C using corresponding antibodies. After several successive washes, membranes were incubated in the presence of a mixture of antibodies and anti-cytokines biotinylated at 4°C overnight. Streptavidin, coupled with HRP, was added on the membranes for 2 h at room temperature. The presence of antibody coupled proteins was revealed by applying ECL (enhanced chemiluminescence) to the membranes, according to the recommendations of the manufacturer. Membranes were then exposed to photosensitive film (Kodak, X-OMAT, AR, USA).

The intensity of chemiluminescence captured on the photosensitive film was measured and recorded. After substracting the backgroud noise, the results were expressed as a ratio of chemiluminescence intensity of experimental versus control spots. The positive control was considered as 1. Less than a −2 ratio value indicated a reduction of the cytokine and a value greater than +2 indicated an increase in cytokine expression.

#### Plasma cytokine array

Plasma cytokine array was performed using different membranes coupled with 100 anti-cytokines antibodies (RayBio Biotin Labeled-based Human Antibody Array) as described above for cell cytokines. Briefly, the membranes were incubated with serum from ovarian cancer patients (diluted 10 times in PBS and BSA 1%). Signal intensities were quantified with a Bio-Imaging System MF-ChemiBis 4.2 (FSVT, Courbevoie, France) and analyzed with Multi Gauge V3.2 software (Fujifilm). For each spot, the net optical density level was determined by subtracting the background optical level from the total raw optical density level.

#### Statistical analysis

Statistical analyses were performed using Prism 4.0 (GraphicPad) or Cricket Graph III (Computer Associates International) software. Parametric statistical analysis (mean ± SEM, linear and exponential regression) was performed using standard methods.

Spearman’s rank correlation coefficients (non-parametric measures) were calculated using R software. Correlation of coefficient significance was determined by a statistical hypothesis test (α=0.05).

## Results

### 

#### Active protein C induces the secretion of cytokines in ovarian cancer cell lines

The *in vitro* cell line, Ovcar-3 and cells of ovarian tumor, share many biological properties. Both express EPCR and PAR-1 ( 18 ) . Both ovarian cancer cells and ovarian cell lines (Ovcar-3 and Skov cells) could be targets for circulating aPC or exogenously added aPC. Since these cells ( 18 ) display certain makers of stem cells (CD117 ^+^ , CD133 ^+^ ), we looked for the influence of aPC on the secretion of physiologically active proteins by the Ovcar and Skov cells and analyzed these proteins using a RayBioTech protein array method.

**Figure 1.**
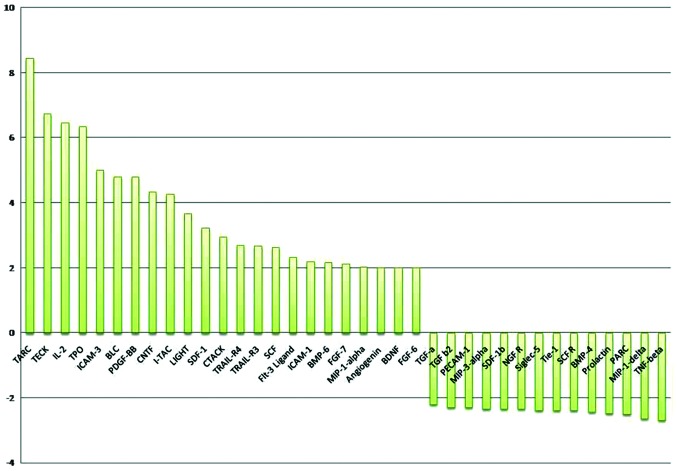
The effect of aPC on the ovarian cancer cell line. In order to analyze the pleiotropic role of active protein C (after treatments of ovarian cell line with aPC for 24 h), we examined the cell supernatants using a protein cytokine array as described in Materials and methods (RayBio ^®^ Human Cytokine Antibody). The ratio of values between cells treated by aPC and non-treated is shown. The positive control was considered as 1. Of the 174 proteins proposed in this test only 23 were upregulated (ratio of cells treated by aPC vs. non-treated cells was >2). They are TARC, TEK, IL-2, TPO, ICAM-3, BLC, PDGF-BB, CNTF, I-TAC, LIGHT, SDF-1α, CTACK, TRAIL-R3, SCF, FLT-3 ligand, ICAM-1, BMP-6, FGF-7, MIP-1α, angiogenin, BDNF and FGF-6 while 14 proteins such as TGFα, TGFβ, PECAM-1, MIP-3α, SDF-1β, NGF-R, Siglec-5, BMP-4, prolactin, PARC, MIP-1δ and TNFβ were downregulated (ratio cells treated by aPC vs. non-treated cells was <2). The ratio situated beyond 4 was taken into consideration for the present study. The results indicate that aPC interacted with tumor cells and activated the secretion of several cytokines such as thymus and activation-regulated chemokine (TARC, CCL17), thymus expressed chemokine (TECK, CCL25), interleukin-2 (IL-2), thrombopoietin (TPO), intercellular adhesion molecule-3 (ICAM-3, CD56), B lymphocyte chemoattractant (BLC), platelet-derived growth factor-BB (PDGF-BB), ciliary neurotrophic factor (CNTF) and T cell alpha chemoattractant (I-TAC) by ovarian cell lines.

Of the 174 proteins proposed in this test only 23 proteins were upregulated (ratio of cells treated by aPC vs. non-treated cells was >2) while 14 were downregulated (ratio of cells treated by aPC versus non-treated cells was <2). When ratio was more than 4 it was considered as a positive response. As presented in Fig. 1 we observed that aPC induced the secretion of several cytokines such as TARC (thymus and activation-regulated chemokine, CCL17), TECK (thymus expressed chemokine, CCL25), IL-2, TPO (thrombopoietin), BLC (B lymphocyte chemoattractant, CXCL13), PDGF-BB (platelet-derived growth factor-BB), CNTF (ciliary neurotrophic factor), I-TAC (T cell alpha chemoattractant CXCL11, also called interferon-inducible T cell alpha) and ICAM-3 (intercellular adhesion molecule 3, CD56) in ovary cell lines at high level (r ≥4). From the data obtained by gene code analysis of all regulated bioactive proteins using DAVID v6.7, we found that these proteins may be involved in cytokine/cytokine-receptor interactions and in immune responses.

#### Evaluation of sEPCR in plasma of ovarian cancer patients

**Figure 2.**
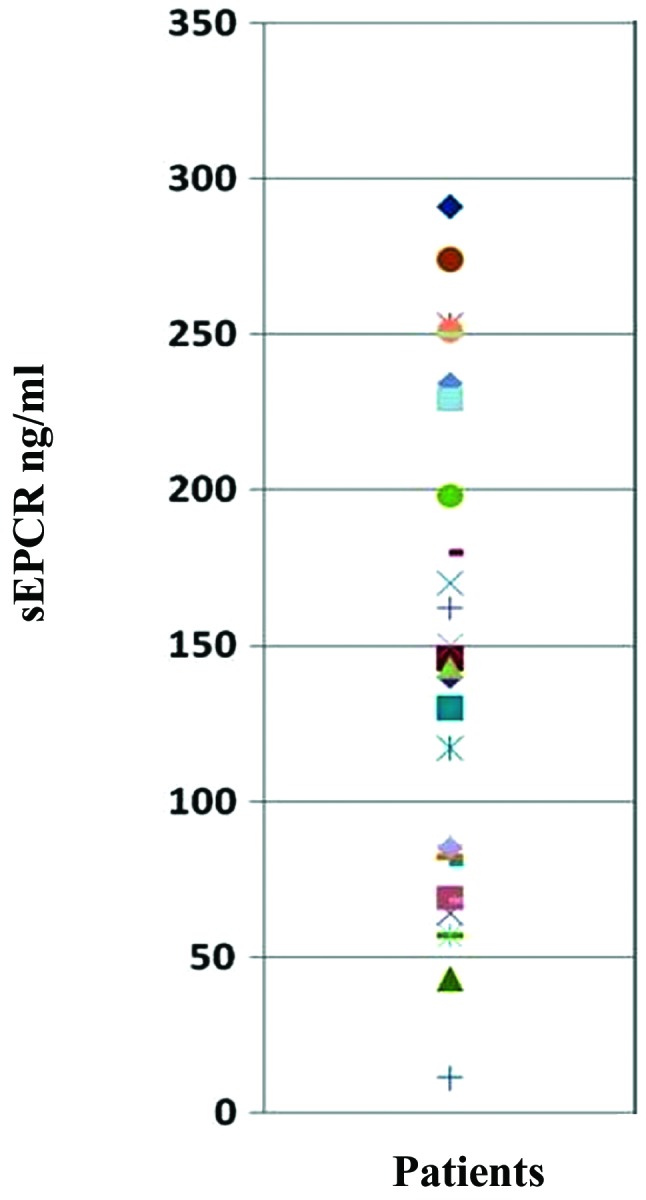
Evaluation of sEPCR in plasma of ovarian cancer patients. Blood (n=33) samples from patients aged >18 years were obtained. Soluble EPCR in plasma fluids was measured using Asserachrom sEPCR immunoassay as recommended by the commercial supplier. All the 33 samples tested were positive for the presence of sEPCR. The base-line value of 100±28 ng/ml was considered as normal. Thus, 60.7% of them exhibited a concentration well about the base-line.

Protein C is involved in septicemia. It can also be trapped by free sEPCR thus rendering it unavailable for thrombotic function. We determined by ELISA the quantity of plasmatic sEPCR in patients diagnosed with ovarian cancer, but asymptomatic. All the samples tested (n=33) were positive for the presence of sEPCR. The base-line value of 100±28 ng/ml was considered as normal. Thus, 61% exhibited a concentration well above the base-line ( Fig. 2 ).

#### Influence of plasma sEPCR on circulating immune cells

To underline the significance of sEPCR in tumor behavior and its influence on circulating immune cells, we analyzed the correlation of sEPCR level in plasma with that of immune cells from blood in 33 patients, using antibodies against CD45, CD45ra, CD3, CD4, CD8, CD25, Foxp3, CD294, CD30, CD56, CD161, CD14, CD16, CD20, CD31, Cd133, CD31, CD10, CD146, IL-21, IL-2, TGFβ, IL-17a, IL10, CD94, CD127, ROR-γ, CD127 and CD29.

**Figure 3.**
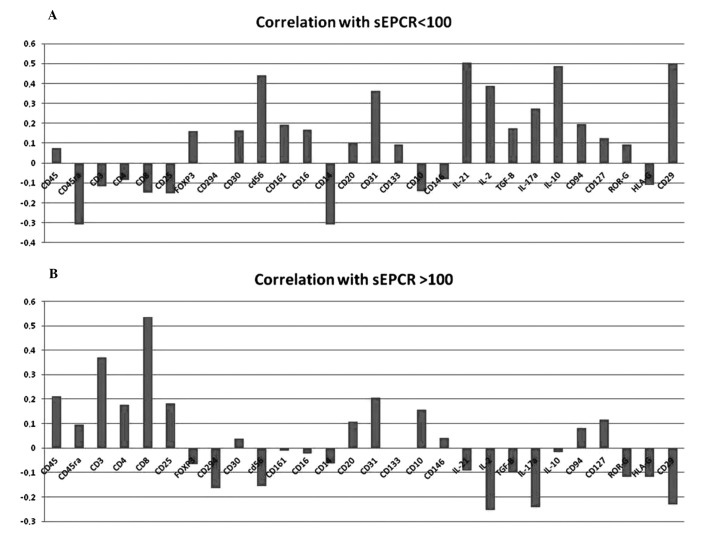
Influence of plasma sEPCR in blood immune cells. Mononuclear cells from 33 patients were separated by Ficoll gradient centrifugation and then stored in 200 *μ* l of DMSO/FCS 50% at −80°C. Phenotyping of immuno-inflammatory cells were performed by flow cytometry the using 30 antibodies as given above. (A) When sEPCR is <100 ng/ml (group 1), plasmatic sEPCR correlated with low intensity of some cell associated markers such as CD45ra, CD3, CD8 and high intensity of with CD56 (NK cells) and cell associated IL-2, IL-10, IL-17a, IL-21 and CD29 markers (r ≤0.40). When sEPCR is >100 ng/ml (group 2), plasmatic sEPCR correlated with high intensity of CD45ra, CD3, CD8, CD25 and low intensity of CD56, IL-2, IL-10, IL-21 and CD29 markers (r ≥0.60). The expression of some cell markers such as CD4, FoxP3, CD20 and CD10 was independent of plasma sEPCR variation (r ≤0.3). A decrease in IL-17a in group-2 (B) was associated with the diminition of CD161 and ROR-γ associated cells. These results indicate that increase of plasma sEPCR was associated with a decrease of effector immune cells in blood circulation.

As indicated in Fig. 3 , the patients were divided into 2 groups: group 1 with sEPCR <100 ng/ml (normal) ( Fig. 3A ) and group 2 with sEPCR >100 ng/ml ( Fig. 3B ). A total of 61% of patients exhibited a concentration of plasma sEPCR well above the base-line (normal plasma level, 100±28 ng/ml). Comparing immune cell phenotypes in patients having a normal level of sEPCR with those having a high level of sEPCR, it was found that sEPCR level was correlated with high intensity of cells expressing CD45ra, CD3, CD8, CD25 and low intensity of cells expressing CD56 (NK cells), CD294 (TH2 cells), IL-2, IL-10, IL-17a (TH17 cells), IL-21 (TH21 cells) and CD29 markers (r ≥0.60). In addition, when level of sEPCR was high, a decrease in IL-17a-expressing cells was associated with decrease of cells expressing CD161 and ROR-γ (RAR-related orphan receptor) involved in the secretion of IL-17 ( Fig. 3 ).

#### Influence of sEPCR on cytokines and interleukins in plasma

**Figure 4.**
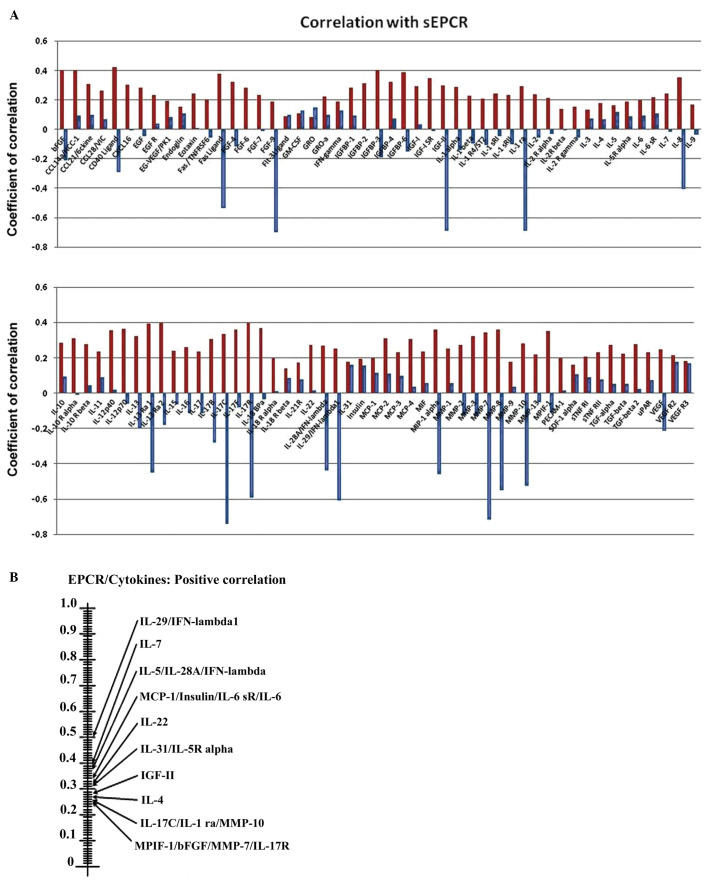
Influence sEPCR on the level of cytokines and interleukins in plasma. (A) We analyzed the correlation between the amount of sEPCR in plasma and a set of 100 biologically active plasma proteins. The membranes were incubated with serum from ovarian cancer patients (10X in PBS albumin 1%). Signal intensities were quantified with a Bio-Imaging System MF-ChemiBis 4.2 and analyzed with Multi Gauge V3.2 software. We compared the significance (r ≥0.60) of plasmatic sEPCR in group 1 (sEPCR <100, blue histograms) and in group 2 (sEPCR >100, red histograms). When plasma sEPCR is >100 ng/ml, the expression of Fas-Ligand, CD 40 ligand, bFGF, FGF-9, IGF-II, IGFbp 3 and 6, IL-ra (receptor antagonist), IL-8, IL-13ra, IL-17b, IL-17c, IL-17R, IL-28A, IL-29, MIP-1, MMP-7, MMP-9, MMP-10 (r ≥0.60) and VEGF (r= 0.40) not only reversed but also increased significantly. (B) The experimental data were further subjected to scrutiny using the Sprearman statistical test. These results confirmed the results shown in (A) and also revealed some additional ones between sEPCR levels and the recently classified type III interferon group.

To understand the influence of sEPCR on plasma cytokines and interleukins, we analyzed, using protein array test the correlation between the amount of sEPCR in plasma (groups 1 and 2) and a set of 100 biologically active plasma proteins. We compared the significance (r ≥0.60) of plasmatic sEPCR in group 1 (sEPCR <100, blue histograms) and in group 2 (sEPCR >100, red histogramms) ( Fig. 4A ).

The experimental data were further subjected to scrutiny using the Sprearman statistical test ( Fig. 4B ). We observed ( Fig. 4A ) that when plasma sEPCR was higher than 100 ng/ml; the expression of Fas-Ligand, CD40 ligand, bFGF, FGF9, IGFII (insulin-like growth factor), IGFbp3 and 6 (insulin-like growth factor binding proteins), ILra (receptor antagonist), IL-8, IL-13ra, IL-17b, IL-17c, IL-17R, IL-28A, IL-29, MIP1 (macrophage inflammatory protein), MMP-7 (matrix metalloproteinase), MMP-9, MMP-10 (r ≥0.60) and VEGF (r=0.40) increased significantly compared to when sEPCR level was below 100 ng/ml.

Certain proteins such as IGFII, IL-13rα, MIP1α and MMP7 have already been proposed as biomarkers for ovarian cancer and particularly those with poor prognosis ( 20 – 22 ) . The Spearman statistical test ( Fig. 3B ), confirmed the results shown in Fig. 3A and also revealed some additional ones between sEPCR levels and the recently classified type III interferon group (IL-28A, and IL-29).

## Discussion

In a recently published work we showed that ovarian cancer cell line Ovcar-3 expressed stem cell markers CD133 ^+^ /CD117 ^+^ (C-Kit) ( 18 ) . Another ovarian cancer cell line Skov also expressed C-Kit (80%), a receptor for stromal growth factor. Similar results were also obtained when ovarian tumors were screened (results not shown). Ovarian tumors and the ovarian cell lines also expressed EPCR and PAR-1. In the present study we analyzed, by a proteins array test, the influence of aPC on the secretion of biologically active proteins.

Our results indicate that aPC induces the secretion of cytokines such as TARC ( 23 , 24 ) , TECK ( 25 ) , IL-2 ( 26 ) , TPO ( 27 ) , BLC ( 28 ) , PDGF-BB ( 29 ) , CNTF (ciliary neurotrophic factor) ( 30 ) , I-TAC ( 31 ) and ICAM-3 ( 32 ) by tumor cells. These pleiotropic cytokines and proteins, believed to play a role in the development of T cells, are in addition chemoattractants for B and T lymphocytes. In light of these observations, we predict that ovarian cancer cells could be targets for circulating protein C, which in turn may be affected by plasma sEPCR.

In the study reported here, we present data on the significance of soluble endothelial protein C receptor (sEPCR) in circulating immune cells and the regulation of cytokines and interleukins in plasma of patients with ovarian cancer.

We investigated the influence of plasma sEPCR on the immune system by characterizing immune cells from 33 ovarian cancer patients as described in Materials and methods. Based on the sEPCR level, group 1 patients, showed high content of CD29, CD31, CD56 and IL-2, IL-10, IL-17a, and IL-21 associated immune cells and low levels of CD45ra and CD14. Surprisingly, statistical analysis showed a positive correlation between sEPCR and CD3, CD8 and a negative correlation between CD56 (NK cells), CD29 (integrin β-1, present on all blood cells), CD294 (TH2 cells), and the immune cells containing IL-2, IL-17a, IL-10 and IL-21. IL-2 is one of a multitude of cytokines produced by lymphocytes and monocytes that trigger a cascade of immune reactions ( 33 ) . IL-10 inhibits the synthesis of pro-inflammatory cytokines such as IFN-γ, IL-2 and TNFα. It has the ability to suppress the antigen-presentation capacity of antigen presenting cells ( 34 ) . The exact role of T helper (Th) 17 cells in malignancy is currently under debate. Th17 plays a protective role against extracellular bacteria and fungi by inducing an inflammatory response ( 35 , 36 ) . Interleukin-21 is a potent immunomodulatory four-α-helical-bundle type I cytokine and is produced by NKT and CD4 ^+^ T cells ( 37 ) . CD294 is a prostaglandin D2 receptor that mediates the pro-inflammatory chemotaxis and is preferentially expressed in CD4 ^+^ effectors T helper 2 (Th2) cells ( 38 ) . These results indicated that in several ovarian cancer patients with high level of sEPCR, there was a decrease of immune cells that are otherwise crucial in innate immune response.

CA125 is currently used and is an important biologic marker for ovarian cancer ( 39 ) . Other plasmatic proteins such as IGFII, IL-13rα, MIP1α and MMP-7 were reported as ovarian cancer biomarkers associated with poor prognosis ( 40 ) . In a recent study we demonstrated a close correlation between increase in sEPCR and CA125 ( 18 ) . However, the correlation between sEPCR and other cytokines, cited above remains yet to be worked out.

A step in this direction was made by undertaking measurements of soluble plasma cytokines, chemokines and interleukins via a proteins array test. All proteins (r ≥0.60) including Fas-Ligand, CD40 ligand, bFGF, FGF9, IGFII, IGFbp3 and 6, ILra (receptor antagonist), IL-8, IL-13rα, IL-17b, IL-17c, IL-17R, IL-28A, IL-29, MIP1α, MMP-7, MMP-9, MMP-10 and VEGF (r= 0.40) increased when plasma sEPCR was higher than 100 ng/ml (group 2). Markers such as IGFII, IL-13rα, MIP1α and MMP-7 correlated well with high amount of plasma sEPCR and CA125 in ovarian cancer.

Results obtained here indicate that sEPCR has an influence on immune cells and plasma cytokine levels. However, how this occurs remains to be clarified. Treatment of blood mononuclear cells (n=5) with aPC (10 *μ* g/ml for 48 h, *in vitro* ) did not modify significantly the immune cell subpopulation as judged by presence/absence of different characteristic markers (results not shown). Further studies are necessary to investigate the effect of aPC on blood mononuclear cells and their cytokine profile of antigen presenting or effector cells.

Activated protein C is a key inhibitor of fibrin formation. Other than hemostastic function, aPC is also known to exert pleiotropic effects, depending on the cell type expressing EPCR. Among others, aPC interferes with the endothelial cell p53 pathway ( 41 , 42 ) . It also promotes endothelial cell proliferation through MAPK and PI3K signaling pathways ( 43 , 44 ) . Cancer cells expressing EPCR may therefore benefit from the cytoprotective effect imparted by APC ( 45 ) . Scheffer *et al* reported a high expression of EPCR in a large panel of tumor cell lines and have interpreted this in light of the EPCR’s role in coagulation ( 16 ) . Beaulieu and Church ( 17 ) claimed that aPC increases breast cancer cell invasion and chemotaxis through EPCR and PAR-1. These sound findings are demonstrative of the importance of EPCR expression and aPC-EPCR signaling pathway in tumor cells.

The overall data suggest that differential expression patterns of chemokines are involved in the specific inflammatory microenvironment of ovarian cancers. In a parallel, but different study (unpublished data) using mRNA-gene array (n=1200) and DAVID analysis of results, we showed the activation of innate immune gene family such as Jak-STAT ( 46 ) and TOLL-like receptor ( 47 ) when the breast cancer MDA-231 cell line was stimulated by activated protein C. These results suggest that plasma aPC/sEPCR can influence plasma cytokine levels in a quantitative and qualitative manner. As a consequence, circulating or intra-tumoral lymphocytes will respond to variations in plasma sEPCR. Future investigation by a randomized study needs to analyze whether all the biomarkers only correlate with severity of the disease.

The above cited cytokines may have a vast potential as well as pleiotropic function. A precise understanding of their involvement in the development of ovarian cancer is critical in developing effective strategies for disease intervention. This is particularly important in the light of a downregulation of NK cells and the immune cells containing interleukin 2, IL-17a (TH17) and IL-21 which suggests the influence of tumor cells on the innate immune system.
